# Axillary extramammary Paget disease in a patient with a history of Birt-Hogg-Dubé syndrome

**DOI:** 10.1016/j.jdin.2024.06.001

**Published:** 2024-07-25

**Authors:** Melissa M. Rames, Eucabeth Asamoah, Rebecca Danhof, Addison Demer

**Affiliations:** a Department of Dermatology, Mayo Clinic, Rochester, Minnesota; b Division of Dermatologic Surgery, Department of Dermatology, Mayo Clinic, Rochester, Minnesota

*To the Editor:* We present a case of an 81-year-old woman with a history of Birt-Hogg-Dubé (BHD) syndrome who was evaluated in dermatology for a new thin tan-to-pink plaque in her left axilla (Fig 1). A shave biopsy from the lesion revealed extramammary Paget disease (EMPD). Total skin examination was otherwise unremarkable. Thorough oncologic work-up was reassuring and included a mammogram, contrast computed tomography of the chest, abdomen, and pelvis, thyroid ultrasound, gynecologic examination, colonoscopy (revealed 4 benign polyps), and review of screening magnetic resonance imaging abdomen from 2 years prior. She was ultimately referred for Mohs micrographic surgery (MMS).

**Fig 1:**
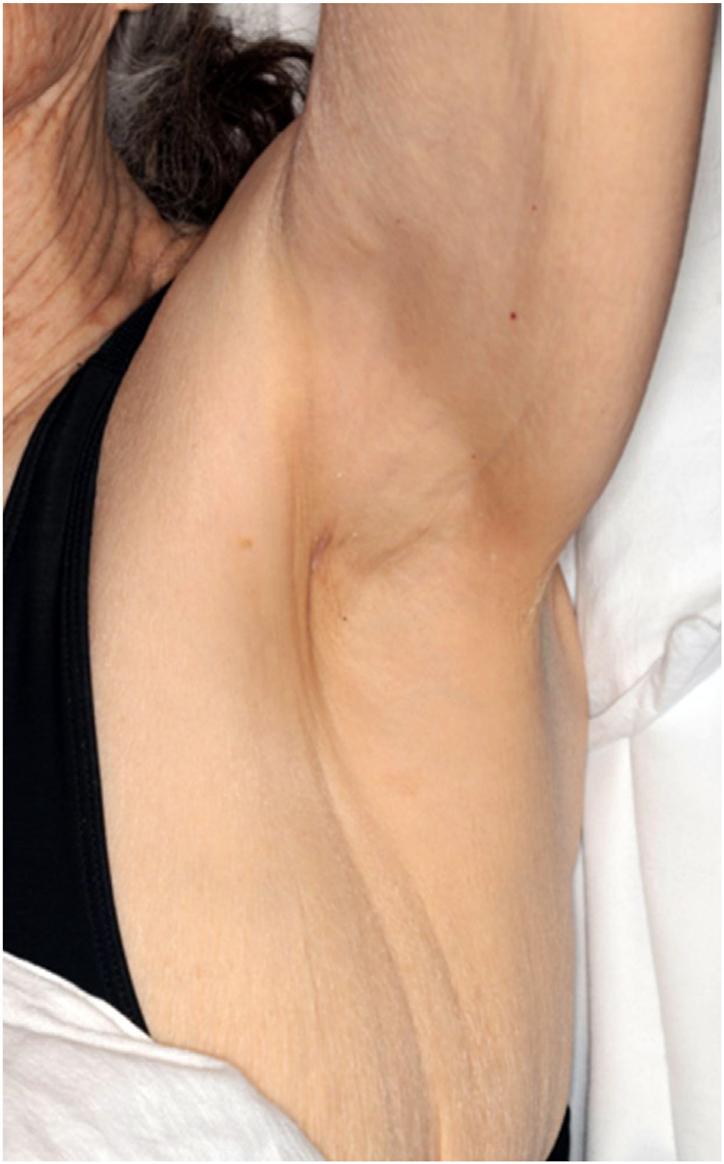
A thin, tan-to-pink plaque on the left axilla, biopsy consistent with extramammary Paget disease.

Before MMS, 13 scouting biopsies of the left axilla were performed to rule out invasive disease and to guide initial surgical margins. The scouting biopsies revealed no evidence of EMPD, and definitive surgical treatment was pursued. The preoperative lesion was small, measuring 1.5 cm × 0.5 cm. The deep and peripheral margins were cleared after one stage of Mohs micrographic excision with Cytokeratin 7 immunohistochemical staining. The postoperative defect was repaired with an immediate layered closure with a postoperative length of 7.2 cm (Fig 2).

**Fig 2:**
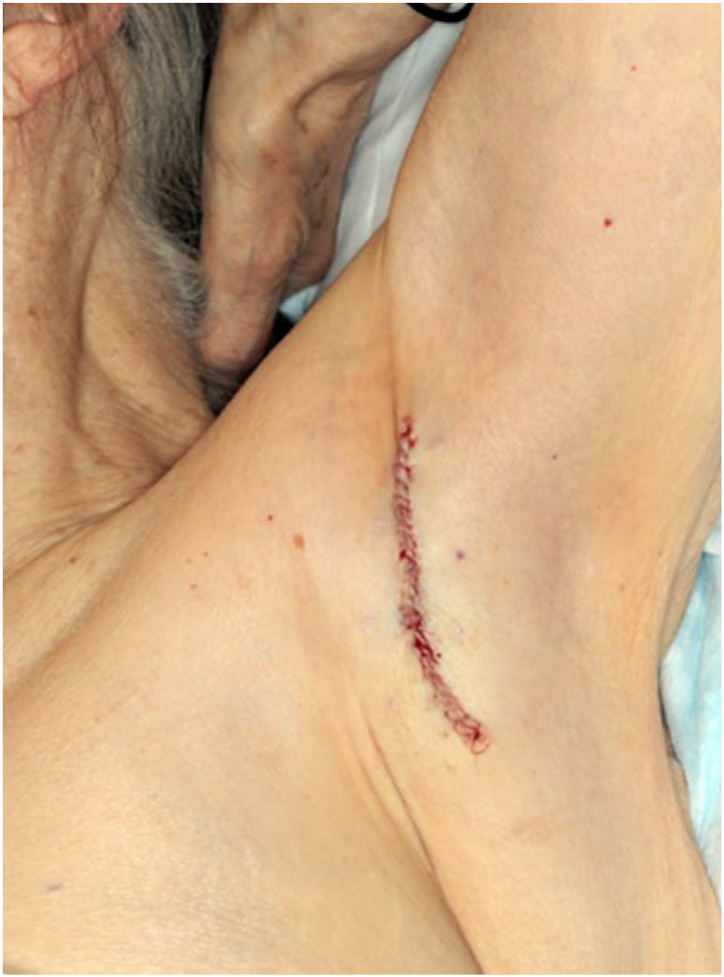
Left axilla after tumor excision and immediate layered closure with Mohs micrographic surgery.

Although BHD and EMPD may both be associated with internal malignancy, there are no reports of these co-occurring. EMPD classically presents as sharply demarcated erythematous plaques with erosions and white scale on apocrine bearing areas such as the groin and axilla.^1^ It commonly arises as a primary adenocarcinoma confined to the epidermis but may present as a secondary extension or manifestation of an underlying carcinoma.^1^ A recent review of patients with axillary EMPD showed 4 patients (25%) had a secondary malignancy—prostate carcinoma, renal cell carcinoma, high-grade prostatic intraepithelial neoplasia, and lung adenocarcinoma (diagnosed 3 years after the EMPD diagnosis).^2^

As subclinical microscopic extension is common, EMPD can challenging to treat.^3^ Surgical excision is the gold standard treatment of EMPD.^3^ MMS or modified MMS (moat method) with intraoperative Cytokeratin 7 immunostaining allows for lower recurrence rates and superior outcomes compared with wide local excision.^3^

BHD syndrome is a rare autosomal dominant genodermatosis due to a germline mutation in the folliculin (FLCN) gene.^4^ The classic cutaneous manifestations include fibrofolliculomas, trichodiscomas, and acrochordons.^4^ Additional cutaneous malignancies reported include leiomyosarcoma, dermatofibrosarcoma protuberans, melanoma, basal cell carcinoma, and squamous cell carcinoma.^4^ The classic systemic manifestations include renal malignancies, pulmonary cysts, and spontaneous pneumothoraces.^4^ However, several additional systemic associations have been reported including medullary thyroid cancer, thyroid adenomas, parotid oncocytomas, basal lung cysts, parathyroid malignancy, colorectal cancer, breast cancer, lung cancer, tonsillar cancer, and others.^4^ In patients with BHD, it is recommended they receive a full skin examination every 6 to 12 months, a lung computed tomography in patients with suspected or risks for pneumothoraces, an annual abdominal/pelvic magnetic resonance imaging starting at age 20 years, an annual review of systems for parotid tumor screening, an annual thyroid ultrasound, and colonoscopies at intervals based off family history.^5^

In conclusion, we report a novel case of an 81-year-old woman with a history of BHD who presented with EMPD of her left axilla; the co-occurrence of BHD and EMPD has not been reported. After a thorough, but unrevealing malignancy work-up, she was treated successfully with one-stage of MMS with Cytokeratin 7 immunohistochemical staining. This case highlights the importance of age and condition appropriate malignancy screening in patients with BHD, including dermatologic examinations at least annually, as well as the importance of ruling out underlying malignancy in all patients who are diagnosed with EMPD.

## Conflicts of interest

None disclosed.
